# Study of Rotation Speed Curve Optimization under the Three-Body Coupling Grinding Mode

**DOI:** 10.3390/mi14061115

**Published:** 2023-05-25

**Authors:** Wei Yu, Binghai Lyu, Qianfa Deng, Chengwu Wang

**Affiliations:** 1 College of Electrical and Information Engineering, Quzhou University, Quzhou 324000, China; 37012@qzc.edu.cn; 2 Key Laboratory of E&M, Zhejiang University of Technology, Hangzhou 310014, China; icewater@zjut.edu.cn; 3 College of Engineering, Zhejiang Normal University, Jinhua 321004, China; cwuwang@126.com

**Keywords:** three-body coupling grinding mode, precision ball, sphericity, rotation angle, grinding uniformity

## Abstract

The three-body coupling grinding mode of a ball ensures the batch diameter variation and batch consistency of precision ball machining based on the principle of ball forming, resulting in a structure that is simply and feasibly controllable. The change in the rotation angle can be jointly determined using the fixed load of the upper grinding disc and the rotation speed coordination of the inner and outer discs of the lower grinding disc. Related to this, the rotation speed is an important index to guarantee grinding uniformity. To ensure the quality of three-body coupling grinding, this study aims to establish the best mathematical control model of the rotation speed curve of the inner and outer discs in the lower grinding disc. Specifically, it includes two aspects. First, the optimization of the rotation speed curve was mainly studied, and the machining process was simulated with three speed curve combinations: 1, 2, and 3. By analyzing the evaluation index of ball grinding uniformity, the results revealed that the third speed curve combination had the best grinding uniformity, and the three speed curve combinations were optimized on the basis of the traditional triangular wave speed curve. Furthermore, the obtained double trapezoidal speed curve combination not only achieved the traditionally verified stability performance but also overcame the shortcomings of the other speed curves. The mathematical model established in this way was equipped with a grinding control system, which improved the fine control ability of the rotation angle state of the ball blank under the three-body coupling grinding mode. It also obtained the best grinding uniformity and sphericity and laid a theoretical foundation for achieving a grinding effect that was close to the ideal circumstance during mass production. Second, via theoretical comparison and analysis, it was determined that the ball shape and sphericity deviation (SPD) were more accurate than the standard deviation (STD) of the two-dimensional trajectory point distribution. The SPD evaluation method was also investigated via the optimization analysis of the rotation speed curve by means of the ADAMAS simulation. The obtained results coincided with the STD evaluation trend, thus laying a preliminary foundation for subsequent applications.

## 1. Introduction

Precision balls, the core and basic components of roundness measuring instruments, gyros, precision bearings, ball screws, ball guiding rails, and precision measuring instruments are currently enjoying high demand and have been widely used in precision machinery, the petrochemical industry, military defense, aerospace, etc. [1,2,3,4,5,6,7]. The precision and batch consistency of spherical balls greatly affect the performance of precision functional parts [8,9,10,11]. At present, precision instrument and precision machine tool industries have great demand for precision balls, resulting in urgent requirements for complete sets of technologies and equipment dedicated for high-precision and high-precision balls that can be processed in batches. To further improve the level of ultra-precision machining technology, developed countries are working hard to study novel methods and processes to achieve new breakthroughs in this field.

The batch processing of precision balls mainly follows the traditional steel ball processing method (V-groove processing method), whereby during which the “spin angle” of the balls must remain constant [12]. For repeated circular processing [13], the processing orientation is changed via the random movement of the circulating groove, which leads to low processing precision and poor batch consistency. High-precision balls are usually selected one by one from the balls processed in batches, which is a difficult procedure in the field of high-precision ball processing in China and abroad [10,11]. Moreover, the degree of production automation is low, and the production process is affected by human factors [14,15,16,17,18,19,20].

**Figure 1:**
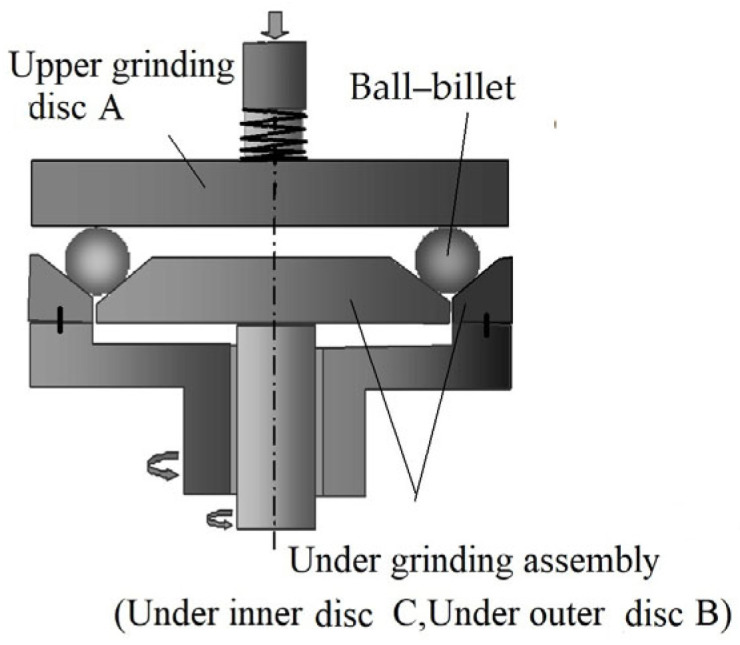
Principle of rotated dual-rotation grinding equipment.

In the current paper, the project team proposed a three-body coupling grinding mode that could realize ultra-precision ball grinding, while ensuring high precision, batch diameter variation, and batch consistency of precision ball processing from the principle of ball forming. Its main principle is shown in Figure 1.

**Figure 2:**
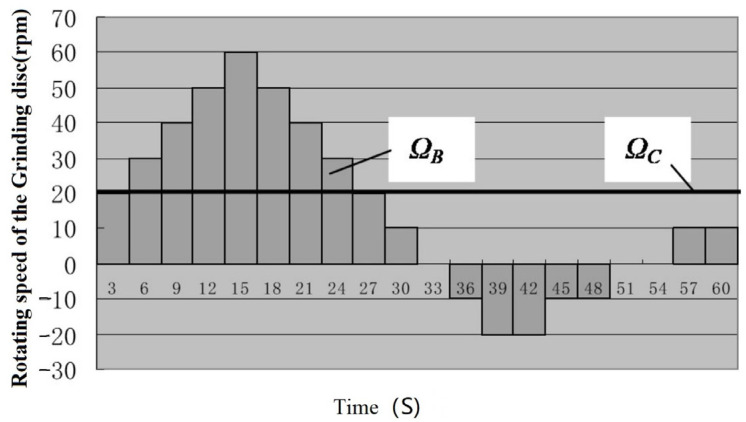
Variation curve of the lower disc speed.

**Figure 3:**
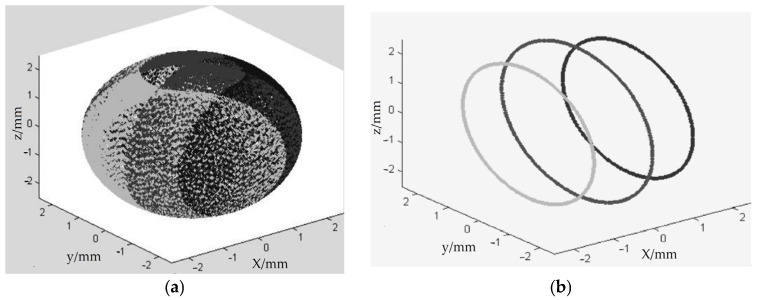
Computer-simulated grinding trajectory distribution [21,22,23,24,25,26]: (**a**) spherical grinding trajectory in three-body coupling grinding mode; (**b**) spherical grinding trajectory in V-groove mode.

The three-body coupling grinding mode entails actively controlling the change in the rotation angle [−90°, 90°] of the ball blank by controlling the change combination of the rotation speeds of the inner and outer discs of the lower grinding disc. In this way, the grinding track covers the whole spherical surface evenly, achieving the uniformity of the grinding track of the machined spherical surface [21]. In turn, this effectively corrects the sphericity error of the spherical surface from the ball-forming principle and improves the machining precision and batch consistency of the entire spherical surface. According to the previous research results, the change curves of the inner disc rotation speed (Ω_C_) and outer disc rotation speed (Ω_B_) of the lower grinding disc were selected, as shown in Figure 2. In addition, the grinding trajectory of the spherical surface under the three-body coupling grinding mode was simulated, as shown in Figure 3a. If the rotation speeds of the inner and outer discs of the lower grinding disc are always the same, this is equivalent to the traditional V-groove grinding mode. The simulated grinding trajectory is shown in Figure 3b. As can be seen, the grinding trajectory of the three-body coupling grinding mode is all over the spherical surface, while the traditional V-groove grinding trajectory is annular. Through the related work completed by the project, the feasibility of the three-body coupling grinding mode has been proven to a considerable extent [21,22,23,24,25,26,27].

However, in practice, there are many factors that affect grinding accuracy, such as the change in motion state and structural error of grinding equipment, which have been detailed in the second paper of this series. In addition, sphericity is mainly related to the variation range and law of the rotation angle of the ball blank during processing. By performing theoretical analysis, the change in the rotation angle can be determined using the fixed load of the upper grinding disc and the rotation speed coordination of the inner and outer discs of the lower grinding disc. To guarantee the ball-forming quality of the three-body coupling grinding mode, the research aims to establish the best mathematical control model of the rotation speed curve of the inner and outer discs of the lower grinding disc, thus ensuring that the precision ball can obtain high precision and batch consistency in a stable and efficient manner under the three-body coupling grinding mode.

## 2. Comprehensive Performance Analysis of Precision Ball Grinding Modes in China and Abroad

To reduce the production cost and improve the precision, efficiency, and consistency of ball machining, many scholars have proposed new machining methods according to the characteristics of ball machining, such as rotation angle actively controlled grinding mode, the three-body coupling grinding mode, and the magnetic float polishing (MFP) mode [21,22,23,24,25,26,27].

### 2.1. Traditional V-Groove Grinding Mode

**Figure 4:**
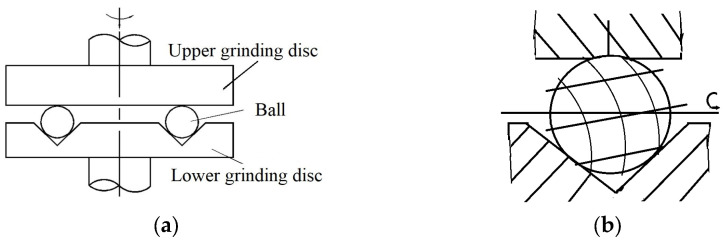
Traditional V-groove grinding mode: (**a**) schematic diagram of the traditional V-groove grinding mode; (**b**) traditional V-groove grinding trajectory line.

The V-groove grinding mechanism is shown in Figure 4a. In this grinding mode, the value of the rotation angle θ only depends on the diameters of the ball and the groove of the lower grinding disc; however, it has nothing to do with the rotation speed of the grinding disc, and it is almost unchanged during the machining process, remaining very small [27,28,29,30,31,32,33,34,35,36,37,38,39,40,41,42,43,44]. The ball can only perform a grinding movement with a “constant relative orientation” (i.e., the angle between the rotation axis and the revolution axis of the ball is constant), and the grinding trajectory lines formed on the surface of the ball by the contact points between the ball and the grinding disc comprise a group of circular rings with the ball rotation axis as the axis, as shown in Figure 4b [21]. The trajectory lines are spread at a very low speed, which is not good for achieving uniform grinding on the spherical surface. Furthermore, it restricts an improvement in the ball precision and machining efficiency.

### 2.2. Rotation Angle Actively Controlled Grinding Mode

**Figure 5:**
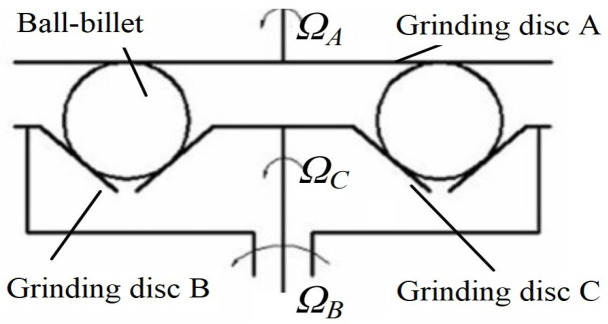
Schematic diagram of active rotation angle control.

**Figure 6:**
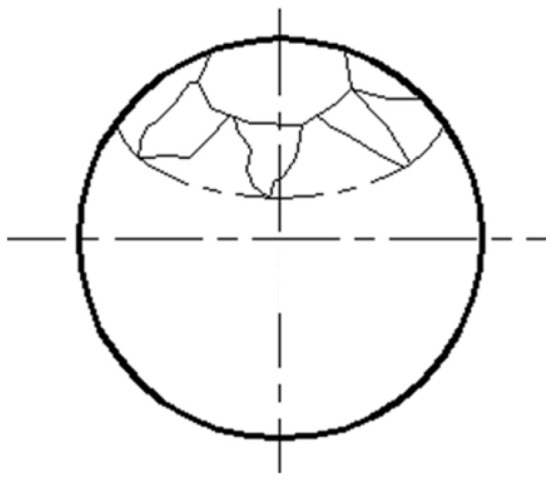
Grinding trajectory with the rotation angle actively controlled.

RyojiKurobe et al. from Kanazawa University, Japan, proposed a rotation angle actively controlled grinding mode (also known as the “coaxial three-disc grinding mode”, as shown in Figure 5) [33,34,35,36,37,38,39,40,41,42,43], in which the lower grinding disc in the V-groove grinding mode was separated at the V-groove, which means that the mechanism consisted of three independently rotatable grinding discs. Furthermore, the rotation angle of the ball blank was adjusted by controlling the rotation speed changes in the three grinding discs. In this grinding mode, the rotation angle θ is not only related to the geometric parameters (e.g., the diameter of the grinding disc and the included angle of the groove of the lower disc) but also to the rotation speed of the grinding disc. By adjusting the rotation speed, θ can be taken in the whole range of [−90°, 90°], and the ball blank can make a “variable relative orientation” grinding movement. The grinding trajectory line is a spatial spherical curve (Figure 6) in which the ball blank rotation axis serves as the axis, covering the whole surface of the ball blank. The grinding disc grinds the ball blank in a “dispersed” manner, which contributes to the uniform and efficient grinding of the ball blank surface. The experimental results reveal that this machining mode can obtain very excellent machining precision and efficiency. However, the complex mechanism of this grinding device limits its application in production.

### 2.3. Three-Body Coupling Grinding Mode

**Figure 7:**
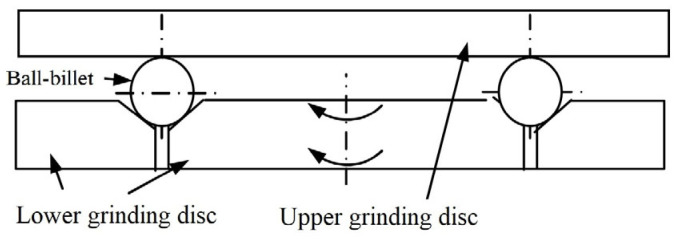
Schematic diagram of the three-body coupling grinding mode.

The rotation angle actively controlled grinding mode, which has a complicated structure, can adjust the rotation angle of the ball to achieve a uniform machining trajectory by controlling the rotation speed of three independent rotary grinding discs. To minimize the complexity of the mechanism and reduce the power source of the grinding equipment, the team of the Ultra-Precision Machining Research Center of Zhejiang University of Technology improved the grinding mode based on the active rotation angle control. They then proposed a three-body coupling grinding mode by combining the process control of the rotation speed change in the grinding disc, whereby the principle of which is shown in Figure 7 [24].

In this grinding mode, the upper grinding disc does not need to rotate. By controlling the rotation speed changes in the inner and outer discs of the lower grinding disc, an adjustment in the relative orientation of the ball’s rotation and revolution axes can be realized; in this way, the grinding trajectory line evenly covers the ball surface, achieving efficient and high-precision grinding of the ball. As the number of drive and transmission devices is reduced from three to two, the structure of the equipment is greatly simplified, and the machining and assembly precision requirements are relatively reduced. As a result, the rotation angle can be continuously changed within the range of [−90°, 90°], and the grinding trajectory can cover the whole sphere, significantly improving the machining precision in the mass production of precision balls. This is highly suitable for the small-batch production of high-precision balls, better meeting the increasing demand for precision balls, and compensating for the shortcomings of the traditional method, including low efficiency and high consumption [40].

**Figure 8:**
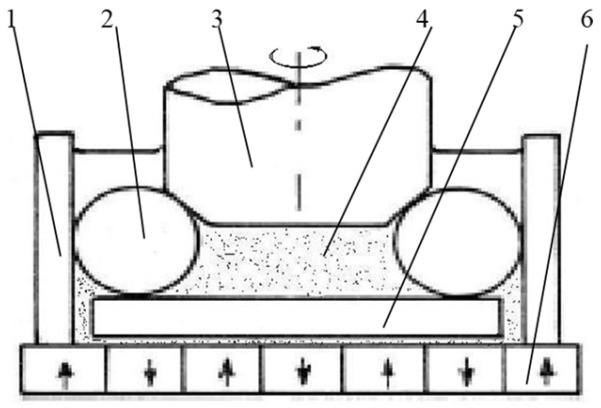
Schematic diagram of the magnetic fluid structure: 1—grinding groove, 2—ceramic ball blank, 3—drive shaft, 4—mixture of magnetic fluid and abrasive, 5—floating plate, 6—magnet.

**Figure 9:**
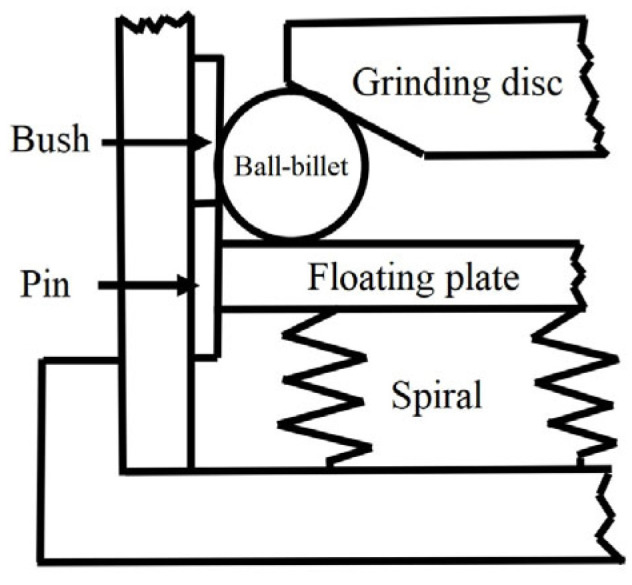
Schematic diagram of the non-magnetic fluid structure.

To realize the efficient grinding of precision balls, Tani and Kawata [37] proposed the MFP method for precision balls in 1984, which greatly enhanced the machining efficiency of precision balls and was later improved by many scholars [44,45]. The principle is shown in Figure 8. A magnetic fluid abrasive is adopted in this method, in which the magnetic fluid pushes the floating plate upward and loads the ball in a given magnetic field. This method is capable of achieving the efficient machining of precision balls at a very high spindle speed (over 10,000 rpm). However, its application is limited due to the high cost and low service life of magnetic fluid. Childs et al. [39,40,41] developed a non-MFP method for producing ceramic balls (Figure 9), in which the magnetic fluid was replaced by a cheap mixture of water and glycerol, the grinding disc was replaced by a resin-bonded diamond grinding wheel, and the self-adaptive supporting force was generated using a coil spring. Their experiment results show that the machining efficiency of this method is equivalent to that of MFP, and the material removal rate can be kept stable during machining [21,22,23,24,25,26,27].

From the above analysis, we can see that the three-body coupling grinding mode has obvious comprehensive advantages in terms of machining precision, machining efficiency, and mechanical structure (Table 1).

## 3. Ball-Forming Principle of Three-Body Coupling Grinding Mode

In this section, the basic principle of precision ball grinding modes was analyzed based on the kinematic equation of the ball blank under the three-body coupling grinding mode [21,22]. This serves as a theoretical foundation for analyzing the influencing factors of the three-body coupling grinding model. In addition, the change laws of the rotation angle and rotation angular speed under different rotation speed combinations were analyzed and compared, along with the envelope of grinding trajectory points on the spherical surface.

### 3.1. Geometric Motion of Ball Blank under the Three-Body Coupling Grinding Mode

In the early research of the group, Wang Z W and Lv B H analyzed the geometric motion of the ball blank under the rotation angle actively controlled grinding mode [21,22]. The geometric motion diagram of the rotation angle actively controlled grinding mode is shown in the figure below.

**Figure 10:**
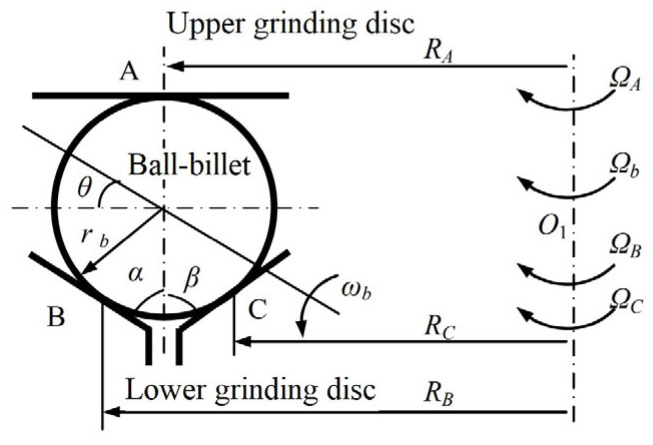
Geometric motion diagram of rotation angle actively controlled grinding mode.

As shown in Figure 10, under the rotation angle actively controlled grinding mode, the machined ball blank came into contact with the grinding disc at three points: A, B, and C in the V-groove, in which discs A, B, and C rotated independently at speeds of Ω*_A_*, Ω*_B_*, and Ω*_C_*, respectively. Under the action of three points, the ball blank produced two kinds of movement: revolution and rotation. Table 2 presents the relevant parameters.

It is assumed that the ball body in this motion is a “real sphere”, that is, any cross-section is a perfect circle, and it does a pure rolling motion. According to the assumption, the speed balance Equation (1) at three contact points A, B, and C is obtained as follows:

(1) $$\left\{\begin{array}{l} R_{A}\Omega_{A}=R_{A}\Omega_{b}-\omega_{b}r_{b}\cos\theta \\ R_{B}\Omega_{B}=R_{A}\Omega_{b}+\omega_{b}r_{b}\sin(\alpha +\theta )\\ R_{C}\Omega_{C}=R_{A}\Omega_{b}+\omega_{b}r_{b}\sin(\beta -\theta ) \end{array}\right.$$

For practically applied grinding discs, *α = β* generally holds. Thus, we have the following:

(2) $$\left\{\begin{array}{l} \tan\theta =\frac{1+\sin\alpha }{\cos\alpha }\cdot \frac{R_{B}\Omega_{B}-R_{C}\Omega_{C}}{R_{B}\Omega_{B}+R_{C}\Omega_{C}-2R_{A}\Omega_{A}}\\ \Omega_{b}=\frac{R_{B}\Omega_{B}+R_{C}\Omega_{C}-2R_{A}\Omega_{A}\sin\alpha }{2R_{A}(1+\sin\alpha )}\\ \omega_{b}=\frac{R_{B}\Omega_{B}+R_{C}\Omega_{C}-2R_{A}\Omega_{A}}{2r_{b}(1+\sin\alpha )\cos\theta } \end{array}\right.$$

When Ω*_A_* = 0 and Ω*_B_* = Ω*_C_* = Ω in Equation (1), the equation is converted into the speed balance Equation (3) under the V-groove grinding mode, and *θ,* Ω*_b_*, and ω*_b_* can be solved similarly, as seen in (4):

(3) $$\left\{\begin{array}{l} 0=R_{A}\Omega_{b}-\omega_{b}r_{b}\cos\theta \\ R_{B}\Omega =R_{A}\Omega_{b}+\omega_{b}r_{b}\sin(\alpha +\theta )\\ R_{C}\Omega =R_{A}\Omega_{b}+\omega_{b}r_{b}\sin(\alpha -\theta ) \end{array}\right.$$

(4) $$\left\{\begin{array}{l} \theta =\tan^{-1}(\frac{r_{b}}{R_{A}}\sin\alpha )\\ \Omega_{b}=\frac{R_{A}\Omega }{r_{b}\cos(1+\sin a)}\\ \omega_{b}=\frac{\Omega }{1+\sin\alpha } \end{array}\right.$$

As shown in Equation (4), the value of the rotation angle *θ* in the V-groove grinding mode only depends on *r_b_* and the grinding disc *R_A_* and has nothing to do with the grinding disc rotation speed Ω [22]. Given that the geometric parameters of the grinding disc are fixed, the rotation angle of the ball body is constant in this machining mode, and because *r_b_ << R_A_*, the rotation angle satisfies θ ≈ 0°. Therefore, the ball blank can only be grinded with a “constant relative orientation”, and the contact point between the ball blank and the grinding disc forms a group of concentric grinding trajectory lines on the surface of the ball blank with the rotation axis of the ball blank as the axis (Figure 3b). Thus, the workpiece cannot be grinded evenly, and the spherical surface cannot be evenly grinded at a quicker pace.

Similarly, when Ω*_A_* = 0 and Ω*_B_ ≠* Ω*_C_* in Equation (1), it is converted into the speed balance equation under the three-body coupling grinding mode, and *θ*, Ω*_b_*, and ω*_b_* can also be solved, as shown in Equation (5):

(5) $$\left\{\begin{array}{l} \tan\theta =\frac{1+\sin\alpha }{\cos\alpha }\cdot \frac{R_{B}\Omega_{B}-R_{C}\Omega_{C}}{R_{B}\Omega_{B}+R_{C}\Omega_{C}}\\ \Omega_{b}=\frac{R_{B}\Omega_{B}+R_{C}\Omega_{C}}{2R_{A}(1+\sin\alpha )}\\ \omega_{b}=\frac{R_{B}\Omega_{B}+R_{C}\Omega_{C}}{2r_{b}(1+\sin\alpha )\cos\theta } \end{array}\right.$$

where $\frac{1+\sin\alpha }{\cos\alpha }$ in tan*θ* is a constant term, which is determined by the geometric angle of the grinding disc itself. In addition, T*R_B_ ≈ R_C_*, that is, tan*θ*, depends on the rotation speed of the grinding disc: $\frac{\Omega_{B}-\Omega_{C}}{\Omega_{B}+\Omega_{C}}$. In theory, its value can be changed in (−∞, +∞), that is, *θ* can vary within [−90°, 90°], which means that it is entirely possible for the dual-rotation mode to make the grinding trajectory point cover the whole spherical surface and the ball blank be uniformly ground by changing Ω*_B_* and Ω*_C_* of the lower grinding discs. The distribution of the grinding trajectories on the spherical surface mainly depends on the rotation angle and rotation angular speed; it has nothing to do with the revolution speed of the ball blank [25,26].

**Figure 11:**
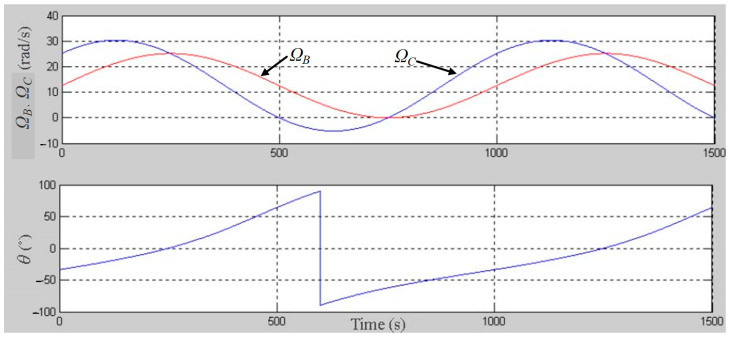
Simulation results of the rotation angle change.

Figure 11 shows the change in the rotation angle *θ* of the ball blank under the simulation conditions (Table 3). As can be seen, under the three-body coupling grinding mode, the grinding trajectory points can fully envelope the spherical surface by adjusting the rotation speed ratio of the inner and outer discs in the lower grinding disc. In turn, this leads to the uniform distribution of the trajectory points and an improvement in the machining precision and batch consistency of the whole ball body.

### 3.2. Characteristics of Rotation Angle under the Three-Body Coupling Grinding Mode

**Figure 12:**
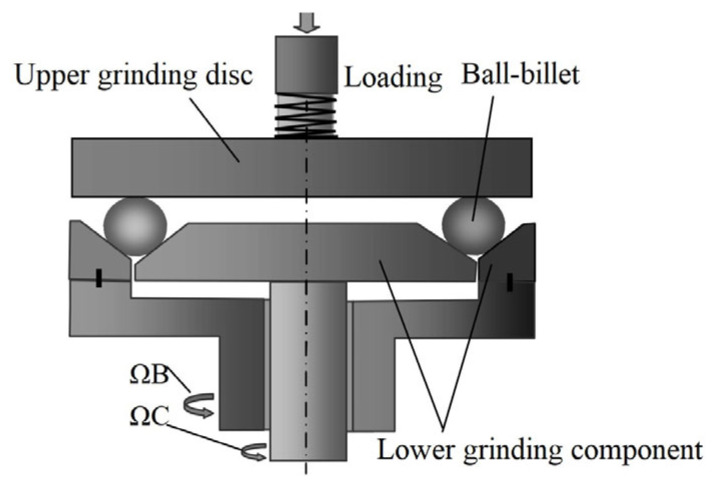
Structural schematic diagram of the three-body coupling grinding mode.

The three-body coupling grinding structure is shown in Figure 12. In the grinding device, grinding is performed via three-point contact among the upper grinding disc, the lower grinding disc, and the ball, in which the upper grinding disc is fixed in the circumferential direction (does not rotate), the lower end surface is the grinding surface, and the pressing device applies a machining load to the ball blank through the upper grinding disc. The lower grinding disc assembly consists of an inner disc and an outer disc, which are, respectively, driven by two motors that can rotate independently via transmission devices. In addition, the conical grinding surfaces on the outer side of the inner disc of the lower grinding disc and on the inner side of the outer disc of the lower grinding disc form a V-groove structure. Driven by the inner and outer discs simultaneously, the ball blank can rotate in two-degree-of-freedom directions. The relative orientation between the rotation and revolution axes of the ball blank can change during grinding by controlling the outer disc of the lower grinding disc and the rotation speed combination of its inner and outer discs. Such an action realizes the ball-forming motion with “variable relative orientations”, thereby uniformly grounding the surface of the ball blank. Moreover, the sphericity deviation can be rapidly corrected so as to improve the machining precision and efficiency. In addition, the equipment structure is simplified, and the machining and assembly precision requirements are relatively reduced, making it highly suitable for the small batch production of high-precision balls [21,22]. Furthermore, the change in the rotation axis of the ball blank is controllable under this grinding mode, and the grinding process is automated. Given that the influence of human factors is excluded, this leads to improved machining consistency and stability.

From the analysis of the structure and working principle of the three-body coupling grinding mode, it can be seen that the rotation angle of the ball blank can vary within [−90°, 90°] during the machining process if the rotation speeds of the inner and outer disc of the lower grinding disc are reasonably coordinated. As such, the grinding trajectory points can be spread all over the spherical surface, which makes it possible to achieve grinding uniformity. Therefore, grinding uniformity can be attained by changing the variation range and law of rotation angle. Furthermore, the influence of the rotation angle change should be evaluated when analyzing the influencing factors of grinding uniformity.

## 4. Simulation Analysis of Speed Curve Optimization

The rotation speed combination of inner and outer grinding discs is the most important factor affecting the distribution of grinding trajectory points. By adjusting the rotation speed combination of the inner and outer grinding discs, the rotation angle and rotation angular speed of the ball can be changed. Moreover, the rotation angle affects the orientation of the grinding area, and the rotation angular speed affects the movement speed of the trajectory points in the grinding area. Thus, various grinding trajectories can be formed under the comprehensive action of the rotation angle and rotation angular speed.

In a certain grinding area of the ball, the grinding path formed by the grinding disk is long, and grinding trajectory points are densely distributed in case of slow changes in the rotation angle, a high rotation speed, and long grinding time. As the rotation angle changes rapidly, the rotation angular speed lowers, and the grinding time is reduced. Furthermore, the grinding path of the grinding disc to the ball in this area is short and the grinding trajectory points are sparsely distributed. Therefore, to obtain better grinding uniformity, the distribution of trajectory points in each grinding area of the ball must be as consistent as possible, which requires the original dense grinding trajectories to become sparse and the original sparse grinding trajectories to become dense.

When improving the distribution of grinding trajectories via an adjustment in the rotation speed combination of the inner and outer discs, which area of the ball the trajectory points are located in must be determined, and any area on the spherical surface must correspond to a range of rotation angle that is unique. When the difference between the outer and inner discs in terms of the rotation speed reaches the upper extremum, the rotation angle reaches the upper extremum as well. When the difference between the outer and inner discs in terms of the rotation speed reaches the lower extremum, the rotation angle also reaches the lower extremum. Then, the density of trajectories in the grinding area is adjusted by changing the change rate in the rotation angle, the rotation angular speed, and the grinding time. To make the original dense grinding trajectories sparse, it is necessary to speed up the change in the rotation angle, reduce the rotation angular speed, and shorten the grinding time, and vice versa. All of the above processes can be realized by adjusting the extreme value of the rotation speed of the grinding disc along with the grinding time, thus changing the distribution state of the grinding trajectories.

The Automatic Dynamic Analysis of Mechanical System (ADAMS) software was adopted to explore the influence of the rotation speed curve combination of the lower inner and outer discs on the grinding uniformity and obtain a better rotation speed combination of the inner and outer discs. Using the program facilitated the simulation of the grinding trajectory under different rotation speed combinations of the inner and outer discs. The MATLAB software was used to quantitatively evaluate the grinding uniformity.

### 4.1. Establishment of the ADAMS Numerical Simulation Model

**Figure 13:**
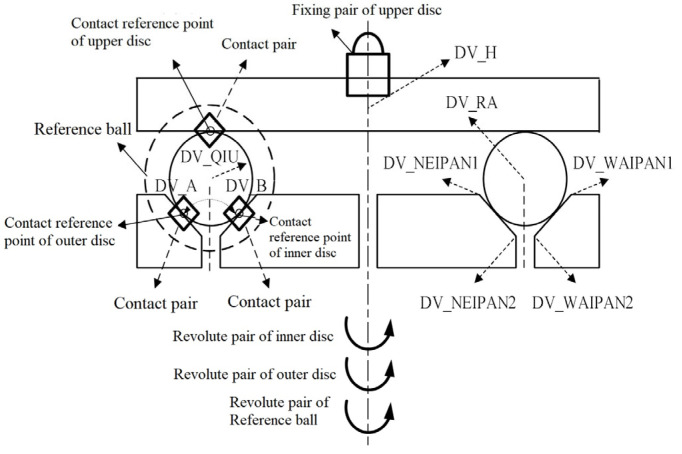
Numerical simulation model of three-body coupling grinding.

Based on the multi-body dynamic analysis software ADAMS developed by MSC, a numerical simulation model of the three-body coupling grinding mode was established, (Figure 13). The variables of parameterization points are displayed in Table 4. This software facilitates the non-linear dynamic analysis and is equipped with advanced numerical analysis technology, which is convenient for the parameter analysis of mechanical system performance and also meets the simulation analysis requirements of the three-body coupling grinding process [45,46]. Therefore, based on this numerical model, the optimal ratio between the mechanical parameters of the grinding disc and the diameter of the machined ball was determined. Then, the relationship between various motion states of the ball and the grinding uniformity during grinding was investigated.

To ensure that the simulation analysis results of the ADAMS numerical model of the three-body coupling grinding mode are as close as possible to the actual machining results, according to the geometric and motion state analysis of the ball formed by the three-body coupling grinding mode and given that the material removal amount and the geometric shape change in the ball are very small in the finish machining stage, the following simulation conditions for the modeling of the three-body coupling grinding mode [45,46,47,48,49,50,51,52] are provided in this study:

1. Ignore the chemical action caused by corrosion or friction of grinding fluid during grinding;
2. Ignore the material removal effect;
3. Take a single ideal true sphere as the analysis object;
4. The contact between the ball and the grinding disc is rigid, without relative sliding.

In addition, the revolution and rotation motion states of the ball center in the ground coordinate system were calculated using the system measurement function in ADAMS software, and after which the revolute pair and driver were added to the reference ball in the model to solve the measurement problem of the motion state at the contact point. After inputting the data of the revolution angular speed of the ball center into the driver, the coordinates of the reference point were finally converted from the ground coordinate system to the ball center coordinate system, thus allowing the grinding trajectory of the ball to be drawn [53,54,55,56,57,58].

**Figure 14:**
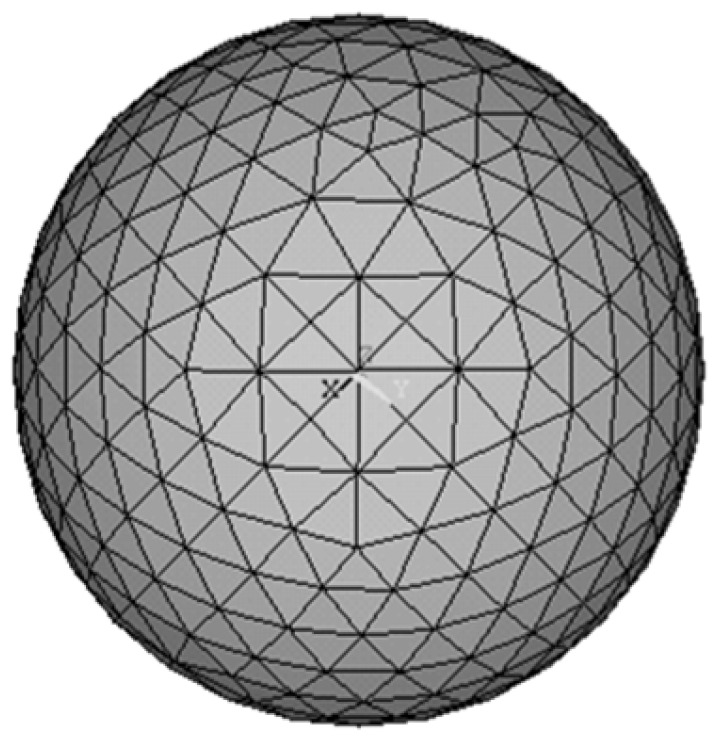
Schematic diagram of triangular grid division.

Using the triangular grid division method, the spherical surface was divided into several triangular areas with the same shape and area to study whether the grinding trajectory points were evenly covered on the spherical surface (Figure 14). Here, the grinding trajectory points were sampled regularly according to the simulation results of the grinding trajectory, and after which the number of grinding trajectory points falling into each triangular area was counted. Then, the standard deviation (STD) of the number of grinding trajectory points in each area was calculated to represent the grinding uniformity. The greater the STD, the worse the distribution uniformity of the grinding trajectory points, and the smaller the STD, the greater the number of trajectory points.

In this model, the single-factor analysis method was used to change the disc diameter and ball radius of the lower inner grinding disc step by step. Then, grinding was simulated, and several groups of grinding trajectory point distribution graphs under different ball radii and disc diameter ratios were acquired. Next, the STD of the number of trajectory points in each area was calculated. In a similar way, the grinding trajectory point distribution graph and the corresponding STD under different groove angles were acquired.

In the simulation of grinding trajectories, it was assumed that the rotation speed of the ball grinding machine was already stable. At the same time, the influence of the rotation speed changed when the starting and shutting down of the grinding trajectory was ignored. However, this is beyond the scope of this study. See the simulation parameters in Table 5.

In this study, three types of speed combinations were selected.

#### 4.1.1. Speed Combination 1

**Figure 15:**
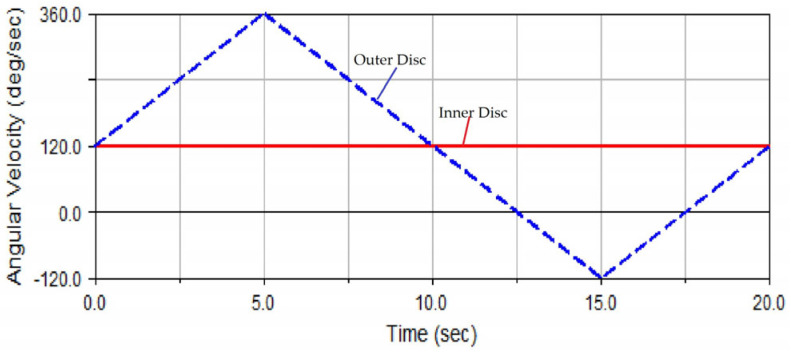
Curve of rotation speed combination 1.

As shown in Figure 15, the curve of rotation speed combination 1 exhibits the rotation speed of the grinding disc used in the traditional dual-rotation grinding mode. This means that the inner disc rotates at a constant speed, while the outer disc rotates at a varying speed according to the triangular wave curve. The rotation speed curves of the two discs are expressed as follows.

In Equation (6), *t* is the time, and *T_c_* is the cycle of the rotation speed change in the grinding disc. The rotation speed ratio *N_t_* varies within [−1, 3]. In addition, Ω_B_ is the rotation speed of the outer disc, Ω_C_ represents the rotation speed of the inner disc, and Ω_0_ is the reference speed.

**Figure 16:**
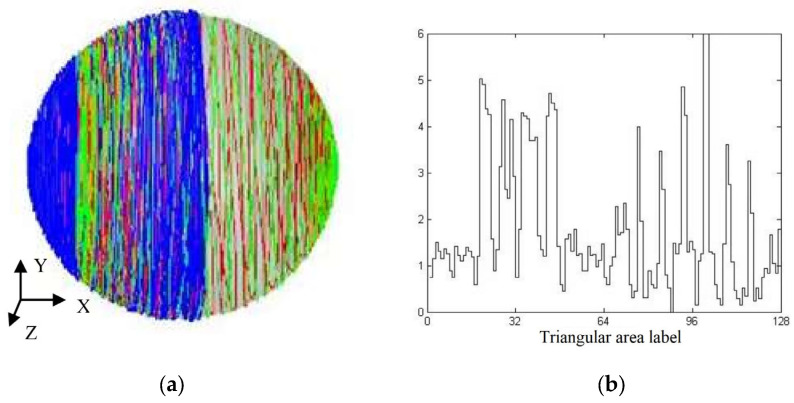
Grinding trajectory and grinding uniformity based on rotation speed curve 1: (**a**) grinding trajectory; (**b**) step diagram of grinding uniformity.

The simulated grinding trajectory and grinding uniformity under the condition of rotation speed curve combination 1 are shown in Figure 16a,b, respectively, in which the STD of grinding uniformity is 1.4409. It could be observed from Figure 16a that the trajectory points were relatively densely distributed in the areas near the left and right ends of the ball. In Figure 16b, regions 0 to 64 correspond to the left hemisphere areas of Figure 4a, while areas 64 to 128 correspond to the right hemisphere of Figure 4a, where area 32 corresponds to the left end point of Figure 4a and region 96 corresponds to the right end point of Figure 4a. At the same time, the numbers of trajectory points in areas 32 and 96 were obviously higher than those in other areas, manifesting that the trajectory points in these two areas were relatively concentrated.

The above phenomenon could be explained as follows: when the rotation speed of the outer disc had just reached the upper extremum of 360 deg/s, the rotation speed of the inner disc reached 120 deg/s, and the rotation speed difference between the outer disc and the inner disc was +240 deg/s—twice that of the rotation speed of the inner disc. In this case, the rotation angle reached 50°, and the grinding point moved near area 96 of the ball, and the rotation speed of the outer disc was also very high; so, the grinding trajectory points near area 96 were concentrated.

When the rotation speed of the outer disc had just reached the lower extremum of −120 deg/s, the rotation speed of the inner disc was 120 deg/s, and the rotation speed difference between the outer and inner disc was −240 deg/s, exactly −2 times that of the rotation speed of the inner disc. In this case, the rotation angle reached −90°. When the grinding point just moved near area 32 of the ball, the rotation speed of the outer disc was also very high at this time; so, the grinding trajectory points near area 32 would be concentrated. However, because the absolute value of the lower extremum (−120 deg/s) of the rotation speed of the outer disc was less than that of the upper extremum (360 deg/s) in area 96, the concentration degree of trajectory points in area 32 was not higher than that in area 96.

Via the above analysis of the grinding trajectory distribution, defects were found in rotation speed combination 1 of the inner and outer discs. This was especially true for the rotation speed curve of the outer disc changing in the form of a triangular wave, with the upper extremum of 360 deg/s and the lower extremum of −120 deg/s just forming the sharp points of two triangles on the curve. Based on the influence of the rotation speed on the grinding trajectory distribution, it could be seen that the absolute values of these two positions of the rotation speed of the outer disc were still too large. Therefore, to obtain better grinding uniformity, the extreme speed of the outer disc in areas 32 and 96 was reduced. At the same time, the difference between the speed of the outer disc and the speed of the inner disc was kept unchanged, and the original rotation angle was maintained, thereby realizing the low-speed grinding of areas 32 and 96. Based on this adjustment strategy, speed combination 1 was improved into speed combination 2.

#### 4.1.2. Speed Combination 2

**Figure 17:**
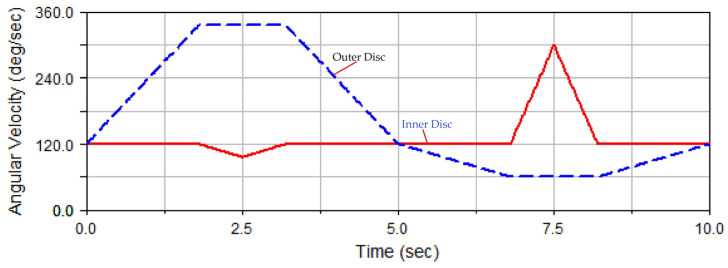
Curve of speed combination 2.

The curve of rotational speed combination 2 is shown in Figure 17. As can be seen, the rotational speeds of both the inner and outer discs are variable, and their expression is shown in Formula (6):

(6) $$N_{t}=\left\{\begin{array}{ll} 1+\frac{4}{0.5T_{c}}t & \;0<t\leq 0.25T_{c}\\ 5-\frac{4}{0.5T_{c}}t & \;0.25T_{c}<t\leq 0.75T_{c}\\ -7+\frac{4}{0.5T_{c}}t & \;0.75T_{c}<t\leq T_{c} \end{array}\right.$$

$$\begin{array}{l} \Omega_{B}=N_{t}\cdot \Omega_{0}\\ \Omega_{C}=\Omega_{0} \end{array}$$

where *t* is the time, *T_c_* is the period of the rotation speed change in the grinding disc, *K*1 and *K*2 are the corresponding width coefficients of the trapezoidal steps, and *N*1 and *N*2 are the height coefficients of the trapezoidal steps. In addition, the speed ratios *N_tB_* and *N_tC_* are the speed ratios of the outer and inner discs, respectively; Ω_B_ is the speed of the outer disc; Ω_C_ is the speed of the inner disc; and Ω_0_ is the reference speed.

**Figure 18:**
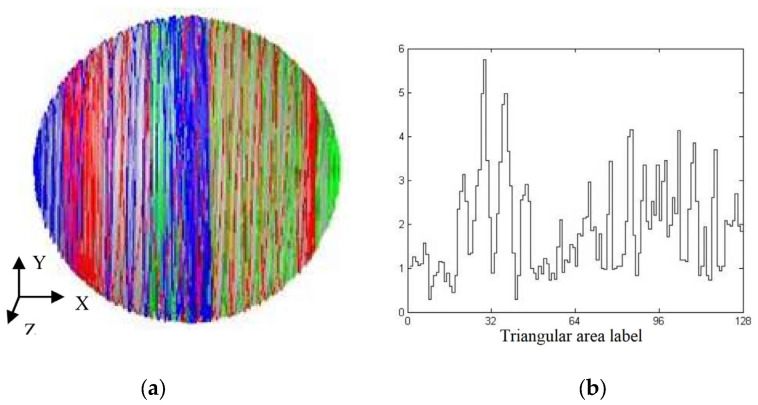
Grinding trajectory and grinding uniformity distribution based on rotation speed curve 2: (**a**) grinding trajectory; (**b**) step diagram of grinding uniformity.

The simulated grinding trajectory and grinding uniformity under the condition of rotation speed curve combination 2 are shown in Figure 18a,b, respectively, in which the STD of the grinding uniformity is at 1.0829. Comparing Figure 16a and Figure 18a, it can be seen that the distribution of grinding trajectories in Figure 18a was obviously more uniform than that in Figure 16a; furthermore, the trajectories in the left and right end areas in Figure 18a were obviously not as concentrated as those in Figure 18a.

By comparing Figure 16b with Figure 18b, it can be seen that the number of trajectory points in area 96 in Figure 18b was obviously less than that in Figure 16b, and the number of trajectory points in area 32 in Figure 18b was also less than that in Figure 16b. In Figure 18b, the number of trajectory points in area 32 was slightly higher than that in area 96; nevertheless, the numbers of trajectory points in areas 32 and 96 were still slightly higher than those in other areas.

The above phenomenon could be explained as follows: when the rotation speed of the inner disc just reached the lower extremum of 100 deg/s and that of the outer disc was 340 deg/s, the rotation speed difference between the outer and inner discs was +240 deg/s, twice the speed of the inner disc. At this time, the rotation angle also reached 50°, and the grinding point just moved near area 96 of the ball. In this case, compared with rotation speed combination 1, the rotation speed of the inner and outer discs evidently decreased. Thus, the grinding trajectory points near area 96 in Figure 18b were more sparsely and uniformly distributed than those in Figure 16b.

When the rotation speed of the inner disc just reached the upper extremum of 300 deg/s and that of the outer disc was 60 deg/s, the rotation speed difference between the outer and inner discs was −240 deg/s, twice the reference speed of 120 deg/s. In addition, the rotation angle also reached −90°. The grinding point just moved near area 32 of the ball. At this time, compared with rotation speed combination 1, the rotation speeds of the inner and outer discs were obviously reduced; so, the grinding trajectory points near area 32 in Figure 18b were more sparsely and uniformly distributed than those in Figure 16b.

$${N_{t}}_{B}=\left\{\begin{array}{cc} \frac{N1}{\left(2.5-K1\right)\cdot T_{C}}\cdot t+1 & \;0<t\leq (0.25-K1)\cdot TC\\ N1+1\;\; & \;0<t\leq (0.25+K1)\cdot TC\\ -\frac{N1}{\left(2.5-K1\right)\cdot T_{C}}\cdot \left(t-0.5T_{C}\right)+1 & \;0<t\leq 0.5T_{C}\\ -\frac{N2}{\left(2.5-K2\right)\cdot T_{C}}\cdot \left(t-0.5T_{C}\right)+1 & \;0<t\leq \left(0.75-K2\right)\cdot T_{C}\\ 1-N2 & \;0<t\leq \left(0.75+K2\right)\cdot T_{C}\\ \frac{N2}{\left(2.5-K2\right)\cdot T_{C}}\cdot \left(t-T_{C}\right)+1 & \;0<t\leq T_{C} \end{array}\right.$$

(7) $${N_{t}}_{C}=\left\{\begin{array}{cc} 1 & 0<t\leq \left(0.25-K1\right)\cdot T_{C}\\ -\frac{2-N1}{K1\cdot T_{C}}\cdot \left(t-0.25T_{C}\right)+1-N1 & 0<t\leq 0.25T_{C}\\ \frac{2-N1}{K1\cdot T_{C}}\cdot \left(t-0.25T_{C}\right)+1-N1 & 0<t\leq \left(0.25+K1\right)\cdot T_{C}\\ 1 & 0<t\leq \left(0.75-K2\right)\cdot T_{C}\\ \frac{\left(2-N2\right)}{K2\cdot T_{C}}\cdot \left(t-0.75T_{C}\right)+1+N2 & 0<t\leq 0.75T_{C}\\ -\frac{\left(2-N2\right)}{K2\cdot T_{C}}\cdot \left(t-0.75T_{C}\right)+1+N2 & 0<t\leq \left(0.75+K2\right)\cdot T_{C}\\ 1 & 0<t\leq T_{C} \end{array}\right.$$

$$\begin{array}{l} \Omega_{B}=N_{t}{}_{B}\cdot \Omega_{0}\\ \Omega_{C}=N_{t}{}_{C}\cdot \Omega_{0} \end{array}$$

The results of grinding uniformity were obtained according to the simulation parameters in Table 6. As shown in Table 7, the STD value of speed combination 1 was 1.4409, while that of speed combination 2 was 1.0829. Generally speaking, the grinding uniformity of speed combination 2 was obviously better than that of speed combination 1, thereby revealing the feasibility and effectiveness of speed combination 2 and the speed optimization strategy in Section 2.3.

**Figure 19:**
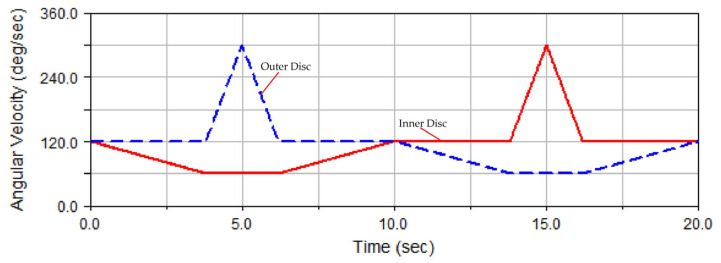
Curve of speed combination 3.

However, as seen through Figure 18b, the fluctuation in the number of trajectory points in each area from 0 to 64 was obviously higher than that in areas 65 to 128, thereby increasing the STD value of the grinding uniformity to some extent. If the fluctuation in the number of trajectory points in each area from 0 to 64 can be reduced to the levels in areas 65 to 128, the STD value of grinding uniformity can be reduced and the grinding uniformity can be improved. A grinding trajectory distribution state corresponds to a rotation speed combination, and in this study, the grinding trajectory distribution state in areas 65 to 128 corresponded to the lower half of rotation speed combination 2. In this study, speed combination 2 was further improved as follows: the upper half of the inner disc speed curve was interchanged with the upper half of the outer disc speed curve, so that the former presented an inverted trapezoidal shape, the latter was triangular, and the lower halves of the inner and outer disc speed curves remained unchanged, thus designing speed combination 3, as shown in Figure 19.

#### 4.1.3. Speed Combination 3

The curve of rotation speed combination 3 is shown in Figure 19, and the rotation speeds of the inner and outer discs were both variable, specifically as shown in Equation (8). In this equation, *t* is time, *T_c_* is the period of the rotation speed change in the grinding disc, *K*1 and *K*2 are the respective width coefficients of the trapezoidal steps, and *N*1 and *N*2 are the corresponding height coefficients of the trapezoidal steps. In addition, the speed ratios *N_tB_* and *N_tC_* are the speed ratios of the outer and inner discs, respectively; Ω_B_ and Ω_C_ are the speed levels of the outer and inner discs, respectively; and Ω_0_ is the reference speed.

**Figure 20:**
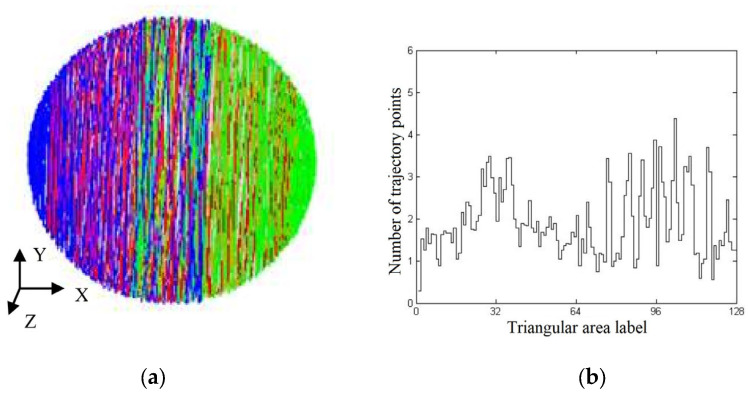
Grinding trajectory and grinding uniformity distribution based on rotation speed curve 2: (**a**) grinding trajectory; (**b**) step diagram of grinding uniformity.

The simulated grinding trajectory and uniformity under the condition of rotation speed curve combination 3 are shown in Figure 20a,b, respectively, in which the STD of grinding uniformity is 0.9748. Comparing Figure 19a and Figure 20a, the two types of grinding trajectories were approximately distributed to a relative extent.

(8) $$\;{N_{t}}_{B}=\left\{\begin{array}{cc} 1 & 0<t\leq \left(0.25-K1\right)\cdot T_{C}\\ \frac{\left(2-N1\right)}{K1\cdot T_{C}}\cdot \left(t-0.75T_{C}\right)+1+N1 & 0<t\leq 0.25T_{C}\\ -\frac{\left(2-N1\right)}{K1\cdot T_{C}}\cdot \left(t-0.75T_{C}\right)+1+N1 & 0<t\leq \left(0.25+K1\right)\cdot T_{C}\\ 1 & 0<t\leq 0.5T_{C}\\ -\frac{N2}{\left(2.5-K2\right)\cdot T_{C}}\cdot \left(t-0.5T_{C}\right)+1 & 0<t\leq \left(0.75-K2\right)\cdot T_{C}\\ 1-N2 & 0<t\leq \left(0.75+K2\right)\cdot T_{C}\\ \frac{N2}{\left(2.5-K2\right)\cdot T_{C}}\cdot \left(t-T_{C}\right)+1 & 0<t\leq T_{C} \end{array}\right.$$

$$\begin{array}{l} \Omega_{B}=N_{t}\cdot \Omega_{0}\\ \Omega_{C}=\Omega_{0} \end{array}$$

Comparing Figure 19b with Figure 20b, the numbers of trajectory points in areas 65–128 were largely identical, but those in areas from 0 to 64 were obviously different. In this section, the trajectory points in Figure 20b were distributed more uniformly than those in Figure 19b. In addition, the number of trajectory points in area 32 in Figure 20b was evidently smaller than that in Figure 19b. The above numerical analysis of grinding uniformity showed that the dense distribution of trajectories under speed combination 3 was inhibited, and the ball grinding uniformity was evidently improved. Therefore, the design strategy of speed combination 3 was feasible and effective.

By exploring the influence of the above three speed combinations on the grinding uniformity, it was found that under a constant speed difference in grinding discs, the grinding uniformity could be improved if the rotation speed of the grinding discs was decelerated. Furthermore, when the speed of the inner disc was higher than that of the outer disc, the grinding uniformity was relatively better. Hence, the curve shape of speed combination 3 was finally selected as being the optimal one.

### 4.2. Simulation of Ball Shape and Calculation of Sphericity Deviation (SPD)

In the actual machining and measurement of balls, their precision is usually evaluated according to the concept of sphericity deviation (SPD). In the national standard (GB/T308-2002), the SPD is defined as the maximum radial distance between the smallest circumscribed sphere and the largest inscribed sphere concentric with the least square sphere in any equatorial plane.

On the basis of the simulation results of the ball grinding trajectory simulation and the quantitative evaluation of the STD of grinding uniformity, the ball shape was simulated and the SPD was calculated in this study to more accurately evaluate the influencing relations of the grinding trajectory and grinding uniformity and the precision of real balls.

**Figure 21:**
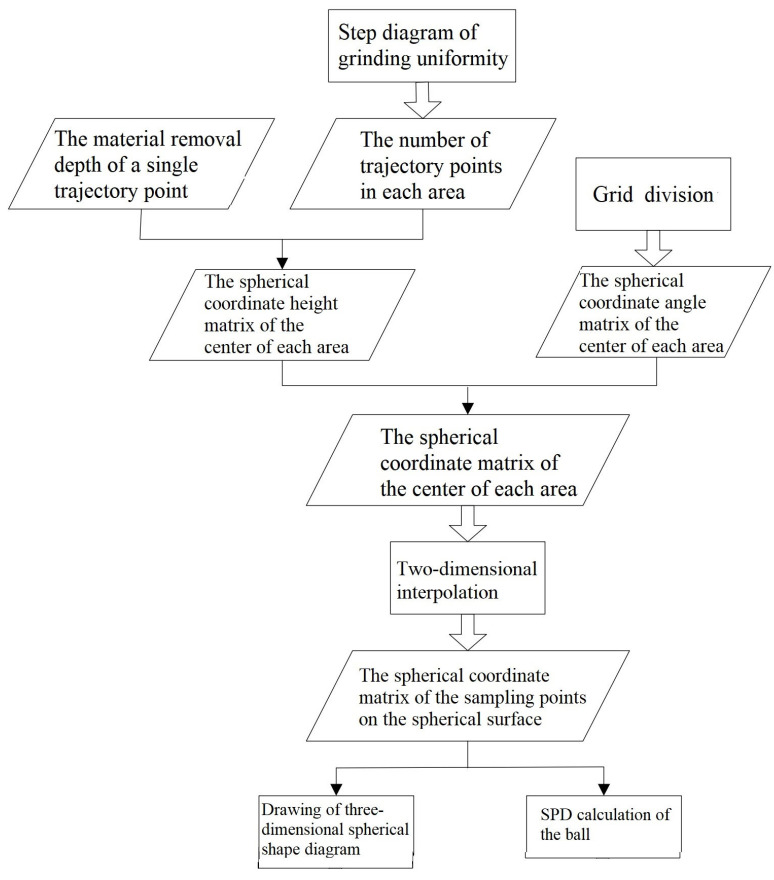
Flowchart of ball shape simulation.

As shown in Figure 21, the steps of the ball shape simulation and SPD calculation can be found below:

1. The material removal depth of a single trajectory point was selected according to the material wear principle (i.e., the material removal depth when the ball contacted the grinding disc once);
2. In accordance with the previous quantitative evaluation of grinding uniformity, the number matrix of the trajectory points in each area was outputted and multiplied by the material removal amount of a single trajectory point to calculate the total material removal depth in each area. For the center of each area, an original spherical coordinate height matrix R_0_ was established and then subtracted by the total material removal depth H in each area to obtain the spherical coordinate height matrix R in the center of each area, considering material removal (i.e., R = R_0_ − H).
3. On the basis of the grid division mentioned above, the horizontal angle THETA and vertical angle PHI of the center of each area in the spherical coordinate system were then calculated.
4. The spherical coordinate angle matrix and height matrix in the center of each area were integrated into one matrix (THETA, PHI, R) to describe the position of the center of each area in the spherical coordinate system.
  - Two-dimensional interpolation was performed on the spherical coordinate matrix of the center of each area using the cubic spline curve interpolation method. The spherical coordinate matrix of the sampling points on the spherical surface was also calculated.
  - According to the spherical coordinate matrix of the sampling points on the spherical surface, a three-dimensional spherical shape diagram was drawn, and the maximum height R_MAX_ was subtracted by the minimum height R_MIN_ in the spherical coordinate matrix of sampling points on the spherical surface to obtain the SPD of the simulated ball shape.

**Figure 22:**
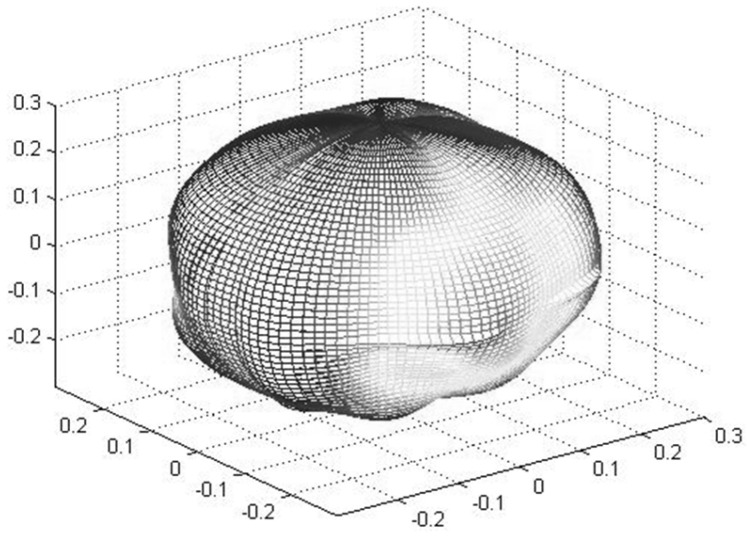
Simulation results of a spherical shape simulation example (unit: μm).

Taking the grinding trajectory during the simulation under the rotation speed combination of the inner and outer discs at 20 s in Figure 19 as an example, Table 6 presents the simulation parameters of the ball shape simulation example. The simulation results are shown in Figure 22, in which the view radius is the product between the ball radius and magnification ratio, that is, 0.3 μm = 15 mm × 2 × 10^−5^. The calculated SPD value is 0.1210 μm.

Table 7 provides the evaluation results of the STD of the grinding uniformity and SPD under three rotation speed combination curves. As shown in the table, the variation trends of the two evaluations were the same and conformed to the simulation results of the grinding trajectory point distribution. Therefore, the evaluation of SPD is theoretically more accurate.

## 5. Conclusions

In this study, the mathematical models and evaluation indexes under three speed curve combinations were established step by step, and the third curve (double trapezoidal speed curve combination) had the best performance and could obtain the best grinding uniformity. STD and SPD were used to evaluate grinding uniformity, and SPD was more accurate. These results were integrated into the control system function of the three-body coupling grinding mode, which improved the fine control ability for the rotation angle state of the ball blank under the three-body coupling grinding mode. The findings also lay a theoretical foundation for achieving a grinding effect that is approximate to the ideal circumstance during practical mass production.

## Figures and Tables

**Table 1:** Comparison of the advantages and disadvantages among various grinding modes.

Grinding Mode	Grinding Efficiency	Grinding Accuracy	Mechanical Structure
Traditional V-groove grinding mode	Low	Low	Simple
Double V-groove grinding mode	High	High	Complex
MFP	Very high	Low	Complex
Rotation angle actively controlled grinding mode	High	High	Complex
Three-body coupling grinding mode	High	High	Relatively simple

**Table 2:** Parameter symbols under the rotation angle actively controlled grinding mode.

Name	Symbol
Contact point between upper and lower grinding discs and the precision ball	A, B, C
Distance of three contact points to the rotary shaft of the lower grinding disc	RA, RB, RC
Rotation speed of the grinding disc	ΩA, ΩB, ΩC
Radius of the ball blank	*r_b_*
Revolution angular speed of the ball body	Ω*_b_*
Rotation angular speed of the ball body	Ω*_b_*
V-groove angle	*A and β*

**Table 3:** Simulation conditions for the grinding trajectory under the grinding conditions of the lower grinding disc.

Rotation speed of grinding disc (rad/s)	Ω_B_ = 100 [sin (0.002πt) + 1]
	Ω_C_ = 100 [sin(0.002πt) + cos(0.002πt) + 1]
Geometric dimensions of grinding disc (mm)	α = 60°
	R_A_ = 100, R_B_ = R_A_ + r_b_cosα, R_C_ = R_A_ − r_b_cosα
Ball radius (mm)	r_b_ = 5

**Table 4:** Meanings and values of design variables.

Name of Design Variable	Meaning
TC	Rotation period
DV_QIU	Ball radius
DV_RA	Distance from the ball center to the rotation axis of the grinding disc
DV_H	Height position of the upper grinding disc
DV_A	External lateral angle of V-groove
DV_B	Internal lateral angle of V-groove
DV_WAIPAN1 DV_WAIPAN2	Design variable of the shape of the lower outer grinding disc
DV_NEIPAN1 DV_NEIPAN2	Design variable of the shape of the lower inner grinding disc

**Table 5:** Simulation parameters.

Simulation Parameter	Time (s)	Reference Speed (d/s)	Number of Grids Divided	Number of Sampling Points
Parameter value	20	120	128	2000

**Table 6:** Simulation parameters of the ball shape simulation example.

Simulation Parameter	Number of Sampled Longitudes	Number of Sampled Latitudes	View (μm)	Ball Radius (mm)	Magnification Ratio
Parameter value	128	64	0.3	15	2 × 10^−5^

**Table 7:** STD and SPD in the step diagram of grinding uniformity.

Grinding Uniformity	Type of Rotation Speed Curve		
	1	2	3
STD (um)	1.4409	1.0829	0.9748
SPD (um)	0.0907	0.0609	0.0473

## Data Availability

Not applicable.
